# Expression of ORF2 protein of TTV for development of EIA system

**DOI:** 10.1186/1471-2334-12-S1-P68

**Published:** 2012-05-04

**Authors:** Shiwani Singh, M Ahmad Ansari, Akanksha Singh, Dhananjay Singh Mankotia, M Irshad

**Affiliations:** 1 Dept. of Laboratory Medicine, All India Institute of Medical Sciences, Ansari Nagar, New Delhi-29, India

## Background/aim

Torque Teno Virus (TTV) is highly prevalent across the world. It is ssDNA virus having 4-5 ORFs of unknown function or antigenicity. To date 40 different genotypes of TTV have been detected. Present study is aimed to determine the antigenicity of ORF2 region of TTV genotype1.

## Methods

ORF2 region of TTV genotype1 (JA20 isolate) was amplified using gene specific primers and cloned in pET19b expression vector for protein expression. Expressed protein was confirmed by western blotting using anti-His antibodies against N-terminal 6XHis tag of expressed protein. Antigenicity of ORF2 was determined by western blot, detecting anti-TTV antibodies in 50 human sera of which, 10 were healthy control negative for TTV-DNA, 10 liver disease cases negative for TTV-DNA and 30 liver disease cases that were positive for TTV-DNA.

## Results

Protein of ORF2 region was successfully expressed and confirmed by western blotting. Anti-TTV antibodies were detected in 25 of 30 (83.3%) cases with liver disease that were positive for TTV-DNA. All 10 cases of liver disease and 10 cases of healthy control, those were negative for TTV-DNA, remains negative for anti-TTV antibodies.

## Conclusion

The results indicate that ORF2 protein is immunogenic, showing low detection of IgG. The reason could be that the protein expressed in this study by TTV genotype1 is producing genotype-specific immunity. However, the study indicates that the antibodies produced during TTV infection does not neutralize or cross-reacts. Further investigations are required to determine whether the protein can be used to develop assay for detection of genotype/genogroup of TTV-infection.

